# Differential Effects of Empagliflozin on Microvascular Complications in Murine Models of Type 1 and Type 2 Diabetes

**DOI:** 10.3390/biology9110347

**Published:** 2020-10-22

**Authors:** Stephanie A. Eid, Phillipe D. O’Brien, Lucy M. Hinder, John M. Hayes, Faye E. Mendelson, Hongyu Zhang, Lixia Zeng, Katharina Kretzler, Samanthi Narayanan, Steven F. Abcouwer, Frank C. Brosius, Subramaniam Pennathur, Masha G. Savelieff, Eva L. Feldman

**Affiliations:** 1 Department of Neurology, University of Michigan, Ann Arbor, MI 48109, USA; steid@med.umich.edu (S.A.E.); phillipe.obrien@gmail.com (P.D.O.); lucyhinder@gmail.com (L.M.H.); jmhayes@med.umich.edu (J.M.H.); fayemend@med.umich.edu (F.E.M.); kkathari@umich.edu (K.K.); sami.narayanan@gmail.com (S.N.); savelief@umich.edu (M.G.S.); 2 Division of Nephrology, Department of Internal Medicine, University of Michigan, Ann Arbor, MI 48109, USA; hyzhang@umich.edu (H.Z.); lixiaze@med.umich.edu (L.Z.); fbrosius@umich.edu (F.C.B.III); spennath@umich.edu (S.P.); 3 Department of Ophthalmology and Visual Sciences, University of Michigan, Ann Arbor, MI 48105, USA; sabcouwe@med.umich.edu; 4 Departments of Molecular and Integrative Physiology, University of Michigan, Ann Arbor, MI 48109, USA

**Keywords:** diabetic neuropathy, diabetic retinopathy, diabetic kidney disease, empagliflozin, oxidative stress

## Abstract

**Simple Summary:**

Type 1 and type 2 diabetes can lead to serious health problems that affect many organs including the nerve, the eye, and the kidney. These health problems can be disabling or even life-threatening, and to date, there are no effective therapies that can slow or prevent their development. We studied the effect of empagliflozin, a medication that reduces blood sugar levels and the risk of cardiovascular disease, on the nerve, eye, and kidney in a side-by-side comparison of type 1 and type 2 mouse models. Empagliflozin had no beneficial effects on disease progression in type 2 diabetic mice, but improved nerve function in the type 1 diabetic model, an effect that was accompanied by reduced markers of oxidative injury. These findings support the concept that different mechanisms contribute to nerve damage in type 1 and type 2 diabetes. Our results further support our view that the concept of one therapy will benefit all complications should be abandoned as we pursue more personalized, tissue-specific, diabetes type-specific treatments.

**Abstract:**

Microvascular complications account for the significant morbidity associated with diabetes. Despite tight glycemic control, disease risk remains especially in type 2 diabetes (T2D) patients and no therapy fully prevents nerve, retinal, or renal damage in type 1 diabetes (T1D) or T2D. Therefore, new antidiabetic drug classes are being evaluated for the treatment of microvascular complications. We investigated the effect of empagliflozin (EMPA), an inhibitor of the sodium/glucose cotransporter 2 (SGLT2), on diabetic neuropathy (DPN), retinopathy (DR), and kidney disease (DKD) in streptozotocin-induced T1D and *db*/*db* T2D mouse models. EMPA lowered blood glycemia in T1D and T2D models. However, it did not ameliorate any microvascular complications in the T2D model, which was unexpected, given the protective effect of SGLT2 inhibitors on DKD progression in T2D subjects. Although EMPA did not improve DKD in the T1D model, it had a potential modest effect on DR measures and favorably impacted DPN as well as systemic oxidative stress. These results support the concept that glucose-centric treatments are more effective for DPN in T1D versus T2D. This is the first study that provides an evaluation of EMPA treatment on all microvascular complications in a side-by-side comparison in T1D and T2D models.

## 1. Introduction

Type 1 (T1D) and type 2 diabetes (T2D) are major public health problems affecting over 463 million individuals worldwide [1]. Up to 50% of diabetes patients suffer from microvascular complications, including diabetic peripheral neuropathy (DPN), diabetic retinopathy (DR), and diabetic kidney disease (DKD) [2,3,4], leading to lower quality of life and higher mortality. DPN affects up to 60% of T1D and T2D patients, and can lead to disability with nonhealing ulcers and eventual lower limb amputation [2]. DR occurs in ≈25% of T2D patients and as many as 50% of T1D patients [3], and can lead to impaired vision and possible blindness. DKD prevalence is ≈25 and ≈30–40% of T1D and T2D patients, respectively, and is the leading cause of end-stage renal disease [4]. Hyperglycemia is a major risk factor for developing microvascular complications, yet despite good glycemic control, diabetes patients, particularly T2D individuals, can still develop diabetes-induced nerve, retinal, and/or renal damage. Therefore, new antidiabetic drug classes are being evaluated for treating microvascular complications.

Empagliflozin (EMPA) is a glucose lowering drug that inhibits the sodium/glucose cotransporter 2 (SGLT2), the transporter responsible for the majority of glucose reabsorption from kidney glomerular filtrate [5]. Inhibiting SGLT2 increases urinary glucose excretion, thereby reducing blood glucose to afford glycemic control. Clinical trials of EMPA demonstrated efficacy in lowering the incidence of cardiovascular complications in T2D patients independent of its glucose lowering ability [6] and have shown reduced progression of DKD. These data led investigators to evaluate whether EMPA may have beneficial effects on diabetic complications. Recent preclinical [7,8,9,10,11,12,13] and clinical [14] studies report that EMPA improves DKD and another SGLT2 inhibitor, canagliflozin, has been found to reduce the numbers of T2D patients with nephropathy progressing to kidney failure or death [15]. In contrast, there are far fewer human clinical trials [16] and animal studies [17] on the effects of EMPA on DR and DPN. Therefore, the therapeutic impact of EMPA on diabetes-mediated nerve, retinal, and, to a lesser extent, renal injury is not well established in T1D versus T2D. Additionally, little is known about the mechanisms underlying the cytoprotective effects of EMPA on diabetic complications, although emerging preclinical data suggests it reduces oxidative stress [12,13].

Here, we investigated the effect of EMPA on DPN, DR, and DKD in a side-by-side comparison in susceptible T1D and T2D mouse models. *Db*/+ mice administered streptozotocin (STZ) were used as the T1D model while *db*/*db* mice were used as our T2D model. Both models capture the salient features of human diabetes, including impaired glucose metabolism and development of microvascular complications [18]. We found that EMPA significantly improved glycemic status in both T1D and T2D mice. Microvascular complications in the T1D model improved to a greater extent than in the T2D model, particularly in terms of nerve function, an effect that was accompanied by a reduction in systemic oxidative stress. Our study highlights the differences among the distinct microvascular complications with respect to both the types of diabetes and treatment responses.

## 2. Materials and Methods

### 2.1. Animals

Male mice aged 4 weeks, either *db*/+ or *db*/*db* on C57BLKS background (BKS.Cg-*Dock^7m^* +/+ *Lepr^db^*/J, Stock No: 000642; Bar Harbor, ME; RRID:IMSR_JAX:000642), were purchased from The Jackson Laboratory (Bar Harbor, ME, USA). Mice were housed in a specific-pathogen-free facility at 20 ± 2 °C and with a 12/12-h light/dark cycle at the Unit for Laboratory Animal Medicine (ULAM) at the University of Michigan. Animals had access to water and food and were monitored daily by veterinary staff. All experimental protocols were approved by the University of Michigan’s Institutional Animal Care and Use Committee (IACUC) (PRO00008116, approved 2 May 2018) and were also in accordance with guidelines by the Diabetes Complications Consortium (http://www.diacomp.org).

### 2.2. Model Choice Metabolic Phenotyping

In order to determine whether C57BLKS *db*/+ mouse as a background strain is prone to metabolic abnormalities similar to those now observed in T1D patients on contemporary treatment [19], a separate cohort of mice, housed as described above, consisted of 5-week old WT (Ctr), *db*/+, and high-fat diet (HFD)-fed (*db*/+ HFD) mice. Body weights were recorded at 5, 8, 12, 16, 24, and 36 weeks of age. Glucose tolerance tests were performed by injecting 1 g/kg body weight glucose (Cat# G-8270, Sigma) IP and serially recording glucose levels at 15, 30, 60 and 120 min, at 16 and 36 weeks of age.

### 2.3. Study Scheme and T1D and T2D Models

**Figure 1:**
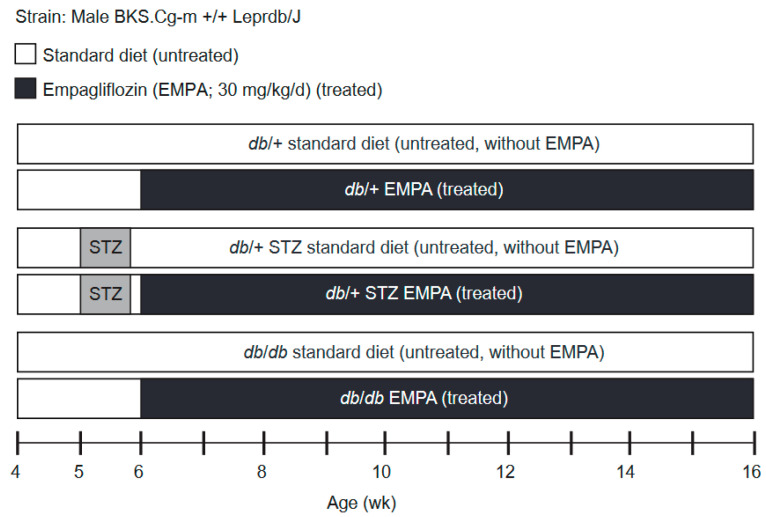
Study scheme and experimental timeline. Type 1 diabetes (T1D) *db*/+ streptozotocin (STZ), type 2 diabetes (T2D) *db*/*db*, and control *db*/+ mice cohorts were randomized at week 6 into treated (Empagliflozin; EMPA, 30 mg/kg per day in chow) or untreated (standard chow) arms. The study lasted 10 weeks, during which body weight and fasting blood glucose (FBG) were recorded every 2 weeks. At week 16, the study conclusion, terminal body weights, FBG, hemoglobin A_1c_ (%HbA_1c_), and plasma lipids (triglyceride and cholesterol levels) as well as microvascular phenotyping (neuropathy (DPN), retinopathy (DR), nephropathy (DKD)) were performed.

The study scheme and experimental timeline are outlined in Figure 1. STZ (Sigma-Aldrich, St. Louis, MO, USA) was prepared in citrate buffer (pH 4.5) and administered for 5 consecutive days (50 mg/kg per day, i.p.) to generate the T1D *db*/+ STZ animals at 5 weeks of age [18]. The T2D model was the homozygous *db*/*db* mouse. A blood glucose level greater than 300 mg/dL was taken as the cut-off for diabetes onset [20]. Treatment commenced at 6 weeks of age, when mice were randomized to control (standard AIN-76A chow only (Research Diets, New Brunswick, NJ, USA)) or EMPA (purchased from Michigan Medicine, University of Michigan), formulated in AIN-76A chow at 30 mg/kg per day. EMPA dose was carefully selected according to publicly available reports published by the Food and Drug Administration (FDA), whereby it was specifically shown that optimal glucose control was observed at 30 mg/mL in male *db*/*db* mice [21]. The same control animals were used as controls in a parallel study examining the therapeutic efficacy of minocycline on diabetic microvascular complications (*manuscript in preparation*). At 16 weeks of age, mice were euthanized by sodium pentobarbital (150 mg/kg, i.p.). Blood was isolated for assessing hemoglobin A_1c_ (%HbA_1c_) and plasma for lipid profiling and oxidative stress measurement. Footpads from the mouse hind paw were removed for evaluating intraepidermal nerve fiber density (IENFD). Renal and retinal tissues were also collected at study termination for DKD and DR phenotyping.

### 2.4. Metabolic Phenotyping

Body weights and fasting blood glucoses (FBG; AlphaTRAK glucometer; Zoetis, Parsippany-Troy Hills, NJ, USA) were recorded every 2 weeks until the experimental endpoint. %HbA_1c_ was quantified at the experimental endpoint by ELISA (Mouse Hemoglobin A1c Assay Kit, cat# 80310; CrystalChem, Elk Grove Village, IL, USA). Serum lipid profiles were assessed at the experimental endpoint by the Mouse Metabolic Phenotyping Center (MMPC) at the University of Cincinnati Medical Center (Cincinnati, OH, USA; www.mmpc.org).

### 2.5. DPN Phenotyping

DPN phenotyping was performed using the criteria established by the Diabetes Complications Consortium (www.diacomp.org/shared/protocols.aspx) using nerve conduction velocity (NCV) and IENFD. NCVs were recorded in the sural sensory nerves and sciatic-tibial motor nerves at study conclusion as previously reported [22]. Briefly, animals were anesthetized with isoflurane (Hospira, Lake Forest, IL, USA) and a heating lamp held their core temperature at 34 °C. Stainless steel needle electrodes (Natus Medical, Pleasanton, CA, USA) recorded sural sensory NCVs at the foot dorsum following antidromic supramaximal stimulation at the ankle, and quantified by dividing the distance with the sensory nerve action potential take-off latency. Sciatic-tibial motor NCVs were recorded at the foot dorsum following orthodromic supramaximal stimulation, first at the ankle then at the sciatic notch. Latencies were measured for each location from the initial onset of the compound muscle action potential. Sciatic-tibial motor NCVs were quantified by subtracting the measured ankle distance from the measured notch distance, and dividing by the difference in ankle and notch latencies.

IENFD was quantified at the experimental endpoint in fixed and stained plantar hind paw footpads [22]. Briefly, IENFD was quantified in hindpaw plantar surface foot pads fixed in Zamboni Fixative (Cat# 1459A, Newcomer Supply, Middleton, WI, USA) overnight at 4 °C then rinsed in 30% sucrose solution in 0.1 M sodium phosphate buffer. Pads were cryoembedded, sectioned (30 μm), stained for the pan-axonal marker PGP9.5 (1:2000; Proteintech, Rosemont, IL, USA), and imaged (Olympus FluoView 500 confocal microscope, 20 × 1.2 objective, 1024 × 1024 pixels, 3.3 μm optical sections). Fiber counts and lengths from three stacked images per mouse were quantified by a blinded investigator and represented as the number of fibers per millimeter.

### 2.6. DR Phenotyping

DR was assessed at study conclusion using protocols from the Diabetes Complications Consortium by DNA fragmentation as a surrogate measure of retinal function. Cleaved apoptotic DNA in the retina was evaluated by ELISA (Cell Death Detection; Roche, Indianapolis, IN, USA) [23]. Briefly, the retina was dissected immediately after the animal was sacrificed and cleaned of all vitreous. The retina was weighed in a mass-tared microtube and homogenized in chilled lysis buffer supplied with the ELISA kit. After vortexing, incubation, and centrifugation, the supernatant was evaluated by the DNA-fragmentation ELISA kit following manufacturer’s instructions. DNA fragmentation, expressed as optical density from the colorimetric ELISA readout, was then reported relative to wet retinal tissue weight (retinal Wt.)

### 2.7. DKD Phenotyping

DKD was measured at study termination by urine albumin-to-creatinine ratio (ACR) and kidney histopathology (mesangial index) as previously reported [22]. ACR was quantified in urine samples from mice on day three of a 3-day stay in a metabolic cage at study end [22,24]. Quantification was performed using an Albuwell M and Creatinine Companion assay, respectively (both Exocell, Philadelphia, PA, USA). Kidney histopathology was scored in left kidney tissue collected from mice after phosphate buffered saline perfusion at study termination [22,24]. Briefly, the left kidney was excised, fixed, sectioned, stained with periodic acid-Schiff (PAS), and imaged using a digital camera. Fifteen glomerular tufts were randomly analyzed per animal and mesangial area was quantified (MetaMorph) by calculating the percentage of PAS-positive area against the total glomerular area.

### 2.8. Nitrotyrosine and Dityrosine Levels

Oxidized tyrosine products (3-nitrotyrosine and *o*, *o’*-dityrosine) in plasma were assessed by isotope dilution liquid chromatography tandem mass spectrometry (LC-MS/MS) [25]. LC-MS/MS was performed by electrospray ionization in positive ion acquisition mode with multiple reaction monitoring on an Agilent 6490 triple quadrupole LC/MS system (Agilent Technologies, Santa Clara, CA, USA). Oxidized tyrosine products levels were normalized to their precursor tyrosine content.

### 2.9. Data Analysis and Statistical Analysis

Data analysis and statistical tests were performed using Prism 7 (GraphPad, San Diego, CA, USA) [26]. Data are expressed as the mean ± standard error of the mean (s.e.m.) and test comparisons were considered statistically significant for *p* < 0.05. Data normality was determined using the Brown–Forsythe F-test. Normally distributed data were compared by a one-way ANOVA *with post hoc* Tukey’s test. Non-normally distributed data were log_2_ transformed and re-evaluated by the Brown–Forsythe F-test. If log_2_ transformation normalized the distribution, data were compared by a one-way ANOVA with post hoc Tukey’s test. If log_2_ transformation did not normalize the distribution, the original data were compared by the non-parametric Kruskal–Wallis with post hoc Dunn’s test.

## 3. Results

### 3.1. Model Choice: db/+ Mice on HFD Are Prone to Metabolic Impairment

Although several chemically and genetically modified T1D animal models exist, they do not accurately mimic disease development in humans [27]. Additionally, some strains, e.g., C57BL/6, do not develop all three microvascular complications [27]. Moreover, humans with T1D now increasingly exhibit obesity and impaired insulin sensitivity [19]. To model this, we hypothesized that a T1D model with a single *db* allele, i.e., STZ *db*/+ mice, would serve as an appropriate T1D model with metabolic disturbances, as now seen in T1D patients on contemporary treatment, including impaired glycemic status and excessive weight gain [19]. Indeed, *db*/+ mice on the C57BLKS background hemizygous for the leptin receptor mutation are prone to metabolic abnormalities [28,29]. Furthermore, STZ *db*/+ mice would be on the same genetic background as our selected T2D *db*/*db* model. To test our hypothesis, we examined the metabolic parameters (Figure S1) of *db*/+ HFD, relative to WT and *db*/+ control mice. On standard diet, *db*/+ mice were slightly more metabolically impaired than WT mice, though not to significance (Figure S1A–C). We found that *db*/+ HFD mice were significantly heavier than their control littermates throughout the study course (Figure S1A) and at study termination (Figure S1B). *db*/+ HFD animals had impaired glucose tolerance at 16 (Figure S1C) and 36 weeks (Figure S1D). These results show that the BKS *db*/+ mouse background upon metabolic challenge, such as HFD, acquires metabolic features now routinely seen in patients with T1D [30] advocating it as a novel and more biologically relevant mouse model for STZ-induced T1D.

### 3.2. EMPA Improves Glycemic Control in T1D and T2D Mouse Models

We conducted a study on STZ-induced *db*/+ T1D and *db*/*db* T2D mice that were placed on standard or EMPA-supplemented chow, together with their respective non-diabetic controls (Figure 1). Throughout the study period, body weight and FBG were recorded every 2 weeks and metabolic and microvascular phenotyping were performed at the experimental endpoint.

**Figure 2:**
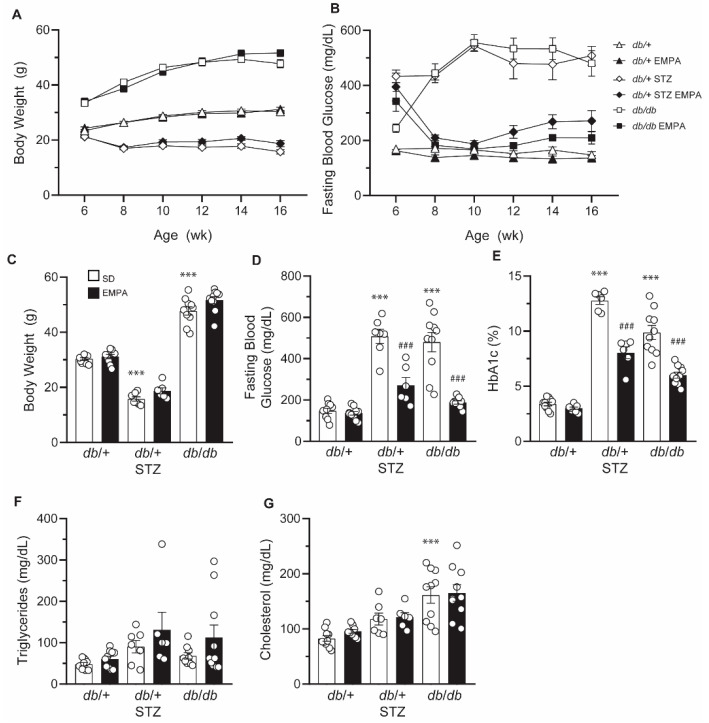
EMPA improves glycemic control in T1D and T2D mouse models. (**A**,**B**) Biweekly body weight measurements (**A**) and FBG levels (**B**) in T1D (*db*/+ STZ), T2D (*db*/*db*), and control (*db*/+) mice with/without EMPA; n = 6–10 mice per group. (**C**–**G**) Terminal body weight measurements (**C**), FBG levels (**D**), %HbA_1c_ (**E**), triglyceride plasma levels (**F**), and cholesterol plasma levels (**G**) in T1D (*db*/+ STZ), T2D (*db*/*db*), and control (*db*/+) mice with/without EMPA; n = 6–10 mice per group; *** *p* < 0.001 for untreated control vs. untreated T1D and T2D; ^###^ *p* < 0.001 for untreated vs. treated T1D or T2D; by one-way ANOVA. Data are expressed as the mean ± s.e.m.

As anticipated, untreated *db*/+ STZ mice displayed a decrease in body weight by 1.9-fold versus *db*/+ controls (Figure 2A,C). The untreated *db*/*db* mice showed a 1.6-fold increase in body weight compared to controls (Figure 2A,C). EMPA did not alter body weight. In T1D and T2D models, FBG levels were elevated and plateaued at around 500 mg/dL (Figure 2B) [20]. After 2 weeks of EMPA administration, both T1D and T2D mice exhibited FBG levels approaching those of *db*/+ controls (Figure 2B). At the terminal time point, FBG was lower in T1D *db*/+ STZ and T2D *db*/*db* mice on EMPA-supplemented chow versus standard diet (Figure 2D). EMPA treatment did not affect the glycemic status in *db*/+ control mice. Terminal measures of hemoglobin A_1c_ (HbA_1c_) were elevated 3.8-fold in *db*/+ STZ mice and 2.9-fold in *db*/*db* mice, compared to *db*/+ controls (Figure 2E). Consistent with the effect of EMPA on glycemic control, terminal %HbA_1c_ was significantly lowered in EMPA-treated T1D and T2D models, although the levels remained significantly higher than controls.

As we and others have shown that dyslipidemia may drive diabetic complications in T2D animal models and patients [31], we next sought to assess the effect of EMPA on circulating lipid profiles (Figure 2F,G). Baseline plasma triglycerides and cholesterol were not significantly altered in untreated T1D *db*/+ STZ versus nondiabetic *db*/+ mice, an effect that was not further impacted by EMPA treatment. While no differences in triglyceride levels were noted in T2D *db*/*db* mice (Figure 2F), cholesterol plasma concentrations were significantly higher than in *db*/+ control animals (Figure 2G). EMPA treatment had no significant effect on these plasma lipid measures in *db*/*db* mice. Additionally, EMPA exerted no effect on the plasma lipid profiles of *db*/+ control animals.

### 3.3. EMPA Ameliorates DPN and DR in T1D but Not T2D Mouse Models

**Figure 3:**
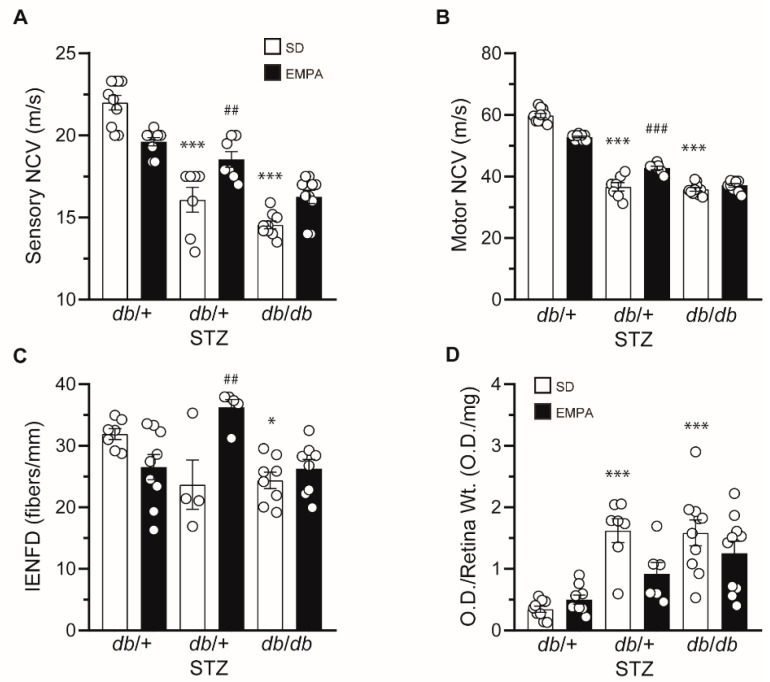
EMPA ameliorates neuropathy and retinopathy in T1D but not T2D mouse models. (**A**–**D**) Terminal sensory nerve conduction velocity (NCV) (**A**), motor NCV (**B**), intraepidermal nerve fiber density (IENFD) (**C**), and terminal retinal apoptosis (**D**) in T1D (*db*/+ STZ), T2D (*db*/*db*), and control (*db*/+) mice with/without EMPA; n = 4–10 mice per group; * *p* < 0.05, *** *p* < 0.001 for untreated control vs. untreated T1D or T2D; ^##^ *p* < 0.01, ^###^ *p* < 0.001 for untreated vs. treated T1D; by one-way ANOVA. Data are expressed as the mean ± s.e.m.

After establishing the effect of EMPA on glucose and lipid measures, we next determined its impact on diabetic complications, by first assessing DPN. As anticipated, both sensory and motor NCVs were decreased in untreated T1D and T2D mice compared to controls (Figure 3A,B). EMPA treatment partially rescued large fiber sensory and motor NCVs in T1D mice, but it had no effect in T2D mice. In control mice, EMPA administration did not significantly influence NCVs. We also assessed small fiber pathology by measuring IENFD loss in the plantar hind paw footpad. When compared to control mice, untreated T1D mice showed a trending reduction in IENFD that did not reach statistical significance (*p* = 0.09) while T2D mice exhibited IENFD loss (Figure 3C), as anticipated [18]. EMPA produced a beneficial outcome in *db*/+ STZ mice, with full IENFD counts consistent with healthy controls, indicating that either EMPA prevented IENFD loss or stimulated small fiber regeneration. In contrast, EMPA did not prevent IENFD loss in *db*/*db* mice. EMPA treatment did not affect IENFD in control mice. Overall, neuropathy phenotyping suggests that EMPA ameliorates DPN in T1D but not T2D mouse models.

We next determined whether EMPA had a beneficial effect on DR. We detected fragmented DNA as a metric of apoptosis in retinal tissue [23]. Both untreated T1D *db*/+ STZ and T2D *db*/*db* mice had increased O.D./Retinal Wt. measures (Figure 3D), over 300% greater than *db*/+ controls, signifying pronounced retinal apoptosis in these models. Although it did not attain statistical significance (*p* = 0.057), EMPA reduced DR by nearly 50% in the T1D model, but had no influence in T2D mice. Similar to DPN, our findings suggest that EMPA may be more effective at improving DR in T1D versus T2D mice.

### 3.4. EMPA Does Not Improve DKD in T1D and T2D Mouse Models

**Figure 4:**
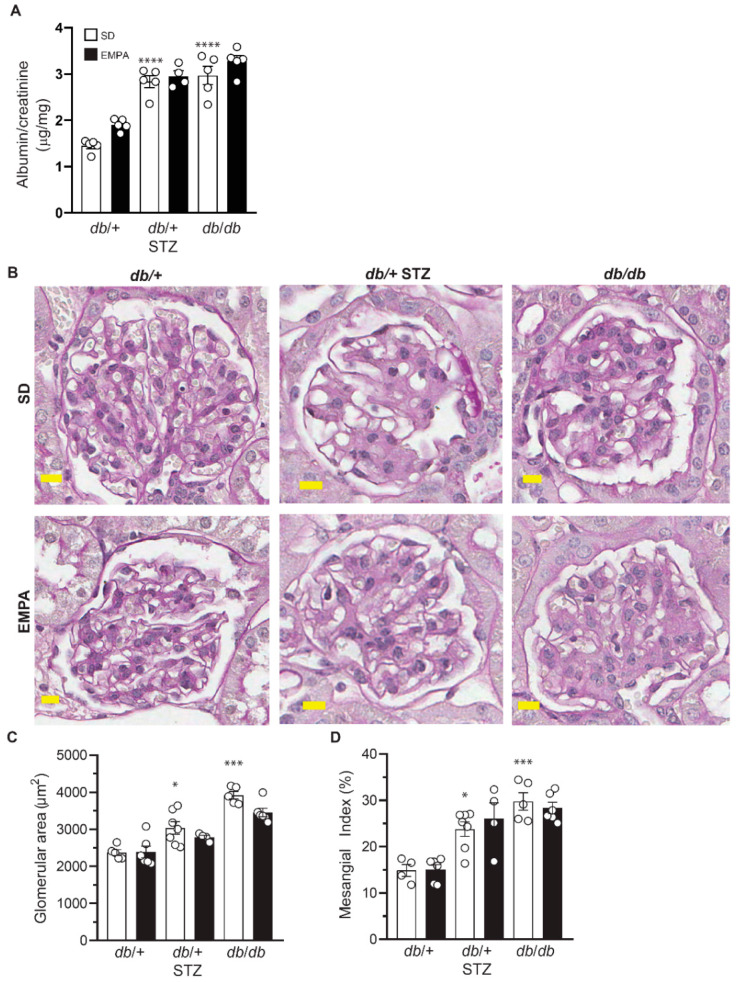
EMPA does not improve nephropathy in T1D and T2D mouse models. (**A**–**D**) Albumin-to-creatinine ratio (ACR) (**A**), representative examples of glomerular periodic acid-Schiff (PAS) staining (**B**), glomerular area (**C**), and mesangial index (**D**) in T1D (*db*/+ STZ), T2D (*db*/*db*), and control (*db*/+) mice with/without EMPA; n = 4–10 mice per group; * *p* < 0.05, *** *p* < 0.001, **** *p* < 0.0001 for untreated control vs. untreated T1D and T2D; by one-way ANOVA. Data are expressed as the mean ± s.e.m.

Next, we analyzed the impact of EMPA on DKD by evaluating albuminuria via ACR (Figure 4A), and the degree of glomerular mesangial expansion by mesangial index (Figure 4B–D). Untreated T1D and T2D mice had substantially and significantly higher ACR versus control animals (Figure 4A) but neither was affected by EMPA treatment. There was a significant increase in cross-sectional glomerular area (Figure 4C) and in mesangial matrix expansion in both T1D and T2D models (Figure 4D) but EMPA administration reduced neither parameter in either model (Figure 4B–D). Overall, T1D and T2D mice both developed renal dysfunction and pathology, but EMPA treatment did not prevent any of these abnormal phenotypes.

### 3.5. EMPA Lowers Systemic Oxidative Stress in T1D Mouse Models

**Figure 5:**
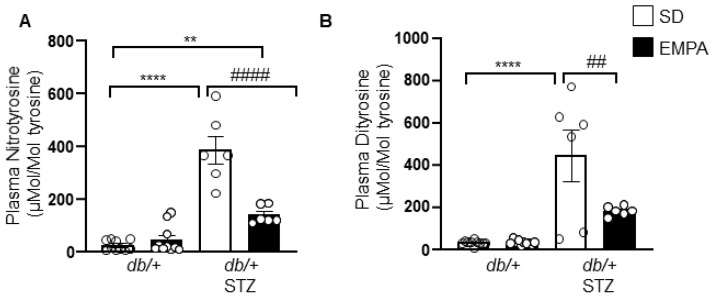
EMPA lowers systemic oxidative stress in T1D mouse model. (**A**,**B**) Plasma levels of nitrotyrosine (**A**) and dityrosine (**B**) in T1D (*db*/+ STZ) and control (*db*/+) mice with/without EMPA; n = 6–10 mice per group; ** *p* < 0.01, **** *p* < 0.0001 for untreated control vs. untreated T1D; ^##^ *p* < 0.01, ^####^ *p* < 0.0001 for untreated vs. treated T1D; by one-way ANOVA. Data are expressed as the mean ± s.e.m.

Oxidative stress is a key pathway of diabetes-induced nerve injury, partly through the oxidation of proteins [32]. One aspect of EMPA cytoprotective mechanisms of action is thought to be through reduced oxidative stress [12,13,33,34]. We next sought to determine whether EMPA exerts its protective mechanisms in T1D-induced DPN in an antioxidative manner. We thus quantified nitrotyrosine and dityrosine levels, highly sensitive markers of protein oxidation in the plasma of T1D mice. Plasma nitrotyrosine and dityrosine levels were significantly higher in *db/+* STZ mice, and decreased upon EMPA treatment (Figure 5A,B). These results indicate that EMPA lowers levels of circulating protein oxidation products, and suggest that improvement in nerve function in the T1D model may occur secondary to EMPA’s potential antioxidant properties.

## 4. Discussion

SGLT2 inhibitors, a new group of drugs that block glucose resorption through an insulin-independent mechanism, i.e., in kidney by blocking SGLT2 transport, are a promising new therapy for diabetes. EMPA, a selective SGLT2 inhibitor with a favorable safety profile, is an important glucose-lowering agent for treatment of T2D. In addition to its antidiabetic actions, studies report beneficial effects of EMPA treatment on cardiovascular complications and renal function in T1D [35,36] and T2D patients [6,14]. In this study, we evaluated the therapeutic efficacy of a 10-week EMPA regimen on diabetic microvascular complications in a side-by-side comparison of T1D and T2D mouse models. EMPA lowered hyperglycemia in T1D and T2D mice and systemic oxidative stress in T1D animals. Mouse models require genetic manipulation to attain robust dyslipidemia [18]. In the current study, lipid profiles in the T1D and T2D animals were modestly altered by disease, and there was no lipid effect of EMPA. EMPA was beneficial to DPN in T1D mice, ameliorating both large and small fiber neuropathy, with a trend toward a salutary effect on DR in T1D animals, which did not reach statistical significance (*p* = 0.057). EMPA had no beneficial effect on microvascular complications in the T2D model.

As anticipated from human trials, EMPA reduced FBG and %HbA_1c_ levels in both our T1D and T2D models, in agreement with other reports that it improves glycemic status in rodents [9,10,11,12,13,33,34]. Herein, we did not observe a change in body weight with EMPA treatment, which is aligned with several other rodent studies [7,10,11,12,13,33,34], but contrasts with reported weight loss in patients [35] and an increase in body weight in two mouse reports [8,9]. We speculate that EMPA’s differential effects on body weight may arise from the various strains employed in the studies, differences between mouse and humans, as well as EMPA’s direct effect on glucosuria and indirect effect on insulin sensitivity [9,37]. Baseline lipid measures were modestly affected by T1D and T2D, a known limitation of mouse models that do not reproduce the lipid profile observed in human diabetes [38,39]. EMPA had no effect on lipid profiles, which mirrors rodent preclinical studies [10,11,12,33,34], but conflicts with human clinical studies. Particularly, EMPA treatment in humans increases LDL levels [40] and decreases triglycerides [41], like all the SGLT2 inhibitors [42].

Hyperglycemia is an established, independent, and prominent risk factor for developing DKD, and glycemic control is the most effective preventive measure against renal failure. Despite restoring glycemic status, DKD did not improve in either T1D or T2D EMPA-treated animals. This is in contrast to the uniformly protective effect of EMPA [14] and other SGLT2 inhibitors on progression of DKD in humans, which appears largely to be independent of glycemic control [15]. There have been less consistent findings of DKD reduction in mouse DKD models treated with EMPA. While most published studies of EMPA effects on DKD in rodents have found a reduction in ACR and mesangial matrix expansion [7,8,11,17], this has not always been the case [9]. The discrepancy among studies may be due to different treatment doses and durations, different rodent models, and diabetes type. Our study also examined animals that had a relatively short duration of diabetes and had developed only a moderate degree of albuminuria and mesangial expansion. Moreover, the EMPA treatment began at an early age (6 wks), and it is conceivable that EMPA treatment at such a young age may have had adverse renal effects that counteracted later beneficial ones [43]. In any case, the lack of effect of EMPA in our mouse studies is not a challenge to the clear-cut benefit of EMPA treatment on DKD progression in humans [44].

To our knowledge, the influence of EMPA on DR in tandem on T1D and T2D animal models has not been previously reported. Our results on a possible lack of EMPA effect on DR in our T2D model are aligned with clinical trial outcomes in T2D patients [16]. While we saw a trend for reducing retinal degeneration in the T1D animal (*p* = 0.057), this did not reach statistical significance. We did not assess vision using functional tests, which could provide different insights into the efficacy of inhibiting SGLT2 on retinal function in T1D and T2D mouse models. Future studies are also needed to understand whether the spectrum of results upon EMPA treatment are secondary to mouse strain and diabetes type [18,27], which can impact the phenotype and treatment response, in addition to treatment dose and duration.

We previously demonstrated in a meta-analysis of multiple clinical cohorts that glucose control effectively manages DPN in T1D patients, but much less so in T2D patients [45]. Here, EMPA similarly improved DPN in T1D but not T2D animals, results that are aligned with human clinical studies. The differential effects of glucose control on DPN in T1D and T2D patients suggest distinct underlying disease mechanisms that have important implications for treatment avenues [2,31,46]. EMPA-controlled blood glucose levels improved DPN in T1D mice, in agreement with another study in T1D rodent models [17]. The degree of DPN improvement in T1D animals in our study is most likely due to glycemic control conferred by EMPA. Conversely, although blood glucose was well regulated in our T2D mice, these animals remained obese with insulin resistance, which is likely one reason EMPA was ineffective [32]. These results agree with our previous findings with *db*/*db* T2D mice with pioglitazone, a PPARγ agonist, where treatment normalized plasma glucose levels but had no effect on large fiber DPN [47]. Our human clinical studies also strongly support obesity, insulin resistance, and dyslipidemia as critical drivers of DPN in T2D [45,48,49].

Oxidative stress is strongly implicated in the pathogenesis of diabetic complications, including DPN [32]. Since EMPA decreases oxidative stress in various tissues, including kidney [12,13], heart [33], and vascular endothelium [34], we speculated EMPA-mediated improvements in nerve function in T1D may occur through antioxidant effects secondary to glycemic control. Consistent with previous reports, our findings also suggest that oxidative stress, as measured by plasma protein oxidation, is a potential mechanism underlying the beneficial effects of EMPA in T1D DPN.

## 5. Conclusions

In summary, we determined whether EMPA exerted a therapeutic effect beyond glycemic control on microvascular complications in T1D and T2D mouse models on the same genetic background. EMPA ameliorated DPN as well as systemic oxidative stress and had a potential modest effect on DR measures in the T1D mouse. Stringent blood glucose regulation helps prevent complications in T1D patients [2] and adjunct EMPA with insulin improves glycemic control [50]. In contrast, EMPA did not appear to exert a statistically significant effect on any complications in the T2D mice. Overall, we observed differential effects of EMPA treatment in T1D and T2D, and within diabetes type on specific tissues, for instance on DPN (effect), DR (possible effect), and DKD (no effect) in T1D. This supports our view that tissue-specific metabolic reprogramming occurs in T1D and T2D [31], and that nervous tissue, eye, and kidney have unique energy requirements and treatment responses, necessitating targeted treatment for diabetic complications [31]. Our study emphasizes the need for future research to address the mechanisms underlying glycemic control in distinct complication-prone tissues. Our study also supports our view that the concept of one therapy will benefit all complications should be abandoned as we pursue more personalized, tissue-specific, diabetes type-specific treatments.

## Supplementary Materials

The following are available online at https://www.mdpi.com/2079-7737/9/11/347/s1, Figure S1: *db*/+ mice fed a HFD gain weight and display impaired glucose tolerance.
