# Supplementary material for: Differential Effects of Empagliflozin on Microvascular Complications in Murine Models of Type 1 and Type 2 Diabetes
Source: Biology (Basel). 2020 Oct 22;9(11):347. doi: 10.3390/biology9110347 (PMC7690408; doi:10.3390/biology9110347)
Supplement: Supplementary file 1 [file biology-09-00347-s001.pdf]

Supplementary Figure 1

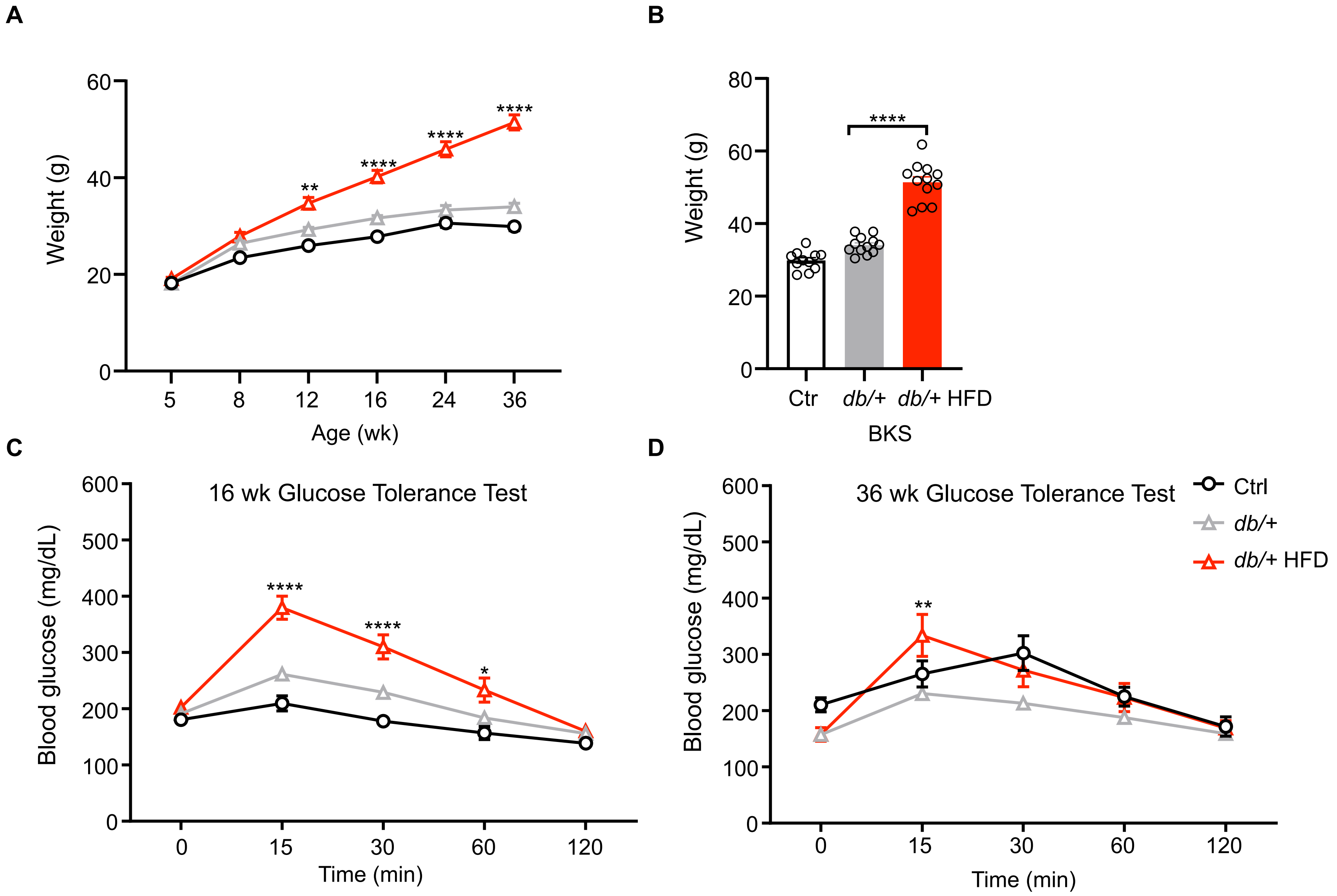

Supplementary Fig. 1. *db/+* mice fed a HFD gain weight and display impaired glucose tolerance. (A-D) Longitudinal body weight measurement (A), terminal body weight measurement (B), glucose tolerance test at 16 weeks (C), and at 36 weeks (D) in *db*/+ mice placed on HFD (*db*/+ HFD), control (*db*/+) and WT control (Ctr) mice; n = 11-12 mice per group; *P < 0.05, **P< 0.01, ***P < 0.001, ****P < 0.0001 vs. *db/*+; by one-way ANOVA. Data are expressed as the mean ± s.e.m.
